# Biochar and Melatonin Partnership Mitigates Arsenic Toxicity in Rice by Modulating Antioxidant Defense, Phytochelatin Synthesis, and Down-Regulating the Transporters Involved in Arsenic Uptake

**DOI:** 10.3390/plants14152453

**Published:** 2025-08-07

**Authors:** Mehmood Ali Noor, Muhammad Umair Hassan, Tahir Abbas Khan, Baoyuan Zhou, Guoqin Huang

**Affiliations:** 1 Research Center on Ecological Sciences, Jiangxi Agricultural University, Nanchang 330045, China; mehmood2017@gmail.com (M.A.N.); muhassanuaf@gmail.com (M.U.H.); tahirsargani@gmail.com (T.A.K.); 2 Key Laboratory of Crop Physiology and Ecology, Institute of Crop Sciences, Chinese Academy of Agricultural Sciences, Beijing 100081, China; zhoubaoyuan@caas.cn

**Keywords:** arsenic, gene expression, melatonin, rice, soil health, yield

## Abstract

Arsenic (As) contamination has significantly increased in recent decades due to anthropogenic activities. This is a serious challenge for human health, environmental quality, and crop productivity. Biochar (BC) is an important practice used globally to remediate polluted soils. Likewise, melatonin (MT) has also shown tremendous results in mitigating metal toxicity and improving crop productivity. Nevertheless, the mechanism of combined BC and MT in alleviating As toxicity in rice (*Oryza sativa* L.) remains unexplored. In this study, we investigated how As affected rice and how the combined BC and MT facilitated As tolerance. The study comprised a control, As stress (100 mg kg^−1^), As stress (100 mg kg^−1^) + BC (2%), As stress (100 mg kg^−1^) + MT (100 µM) and As stress (100 mg kg^−1^) + BC (2%) + MT (100 µM). Arsenic significantly decreased rice growth and yield by increasing electrolyte leakage (EL), malondialdehyde (MDA), and hydrogen peroxide (H_2_O_2_). Co-applying BC and MT substantially enhanced rice growth and yield by increasing chlorophyll synthesis (48.12–92.42%) leaf water contents (40%), antioxidant activities (ascorbate peroxide: 56.43%, catalase: 55.14%, peroxidase: 57.77% and superoxide dismutase: 57.52%), proline synthesis (41.35%), MT synthesis (91.53%), and phytochelatins synthesis (125%) nutrient accumulation in rice seedlings and soil nutrient availability. The increased rice yield with BC + MT was also linked with reduced H_2_O_2_ production, As accumulation, soil As availability, and an increase in *OsAPx6*, *OsCAT*, *OsPOD*, *OsSOD OsASMT1*, and *OsASMT2* and a decrease in expression of *OsABCC1*. Biochar + MT enhanced residual OM- and Fe, ((Fe_2_As) and Mn (Mn_3_(AsO_4_)_2_) bound forms of As leading to a substantial increase in rice growth and yield. Thus, the combination of BC and MT is an eco-friendly approach to mitigate As toxicity and improve rice productivity.

## 1. Introduction

Heavy metals (HM) pollution is a significant threat to crop productivity and soil quality [1]. Recently, soil contamination with toxic metals has substantially increased due to fossil fuels, mining, smelting, poor waste management, synthetic fertilizers, and improper handling of wastewater [2]. These heavy metals pose serious challenges to human health and crop production [2]. Arsenic (As) is one of the most prevalent toxic metalloids that negatively affects crop production and human health. It is a hazardous metalloid due to its carcinogenic characteristics, and it is affecting the health of 94–220 million people globally [3]. Arsenic naturally occurs in soil; however, human activities have significantly increased its concentration in soil [4]. Arsenic present in soil and water triggers many diseases, including cardiovascular failure, neurological disorders, and skin cancer [5]. Arsenic decreases seed germination, seedling growth, and biomass production [6] by causing oxidative stress, membrane damage, and inhibiting chlorophyll synthesis [7]. Arsenic toxicity also decreases chlorophyll synthesis, stomatal conductance, and carbon assimilation, and induces excessive reactive oxygen species production [6,8]. Additionally, As induces metabolic changes and impairs photosynthetic pigments, thereby decreasing plant growth [9]. Therefore, it is essential to develop measures to counter its toxic impacts and prevent its entry into the human food chain.

Globally, different strategies, including phytohormones, carbon-based products, and phytoremediation, are used to remediate polluted soils. Recently, biochar (BC) has emerged as a key agent in mitigating the toxic impacts of HM and metalloids [10]. Biochar is considered “black gold” with appreciable potential to adsorb metals and decrease their availability [11]. Biochar substantially immobilizes toxic metals [12] due to its large surface area with appreciable functional groups. Biochar applied to As-contaminated soil improves chlorophyll synthesis, and gas exchange traits by decreasing ROS production and lipoxygenase activity, and increasing antioxidant activities, and osmoprotectant synthesis [13].

Recently, researchers have explored integrating BC with other amendments to mitigate the effects of toxic metals. Biochar is used with organic amendments, microbes, and phytohormones for mitigating HM toxicity. Biochar combined with phytohormones has emerged as a promising technique to remediate polluted soils. Melatonin (MT) is a signaling molecule with appreciable potential to mitigate adversities of abiotic stresses [14]. Melatonin improves root development, and plant growth, and decreases reactive oxygen species (ROS) production in plants facing stress conditions [15]. Melatonin also protects the plants from toxic metals by affecting their transportation, absorption, and sequestration [16]. It also increases stress tolerance by decreasing the uptake of toxic metals, increasing nutrient uptake [17], and endogenous MT biosynthesis [18,19].

Rice is a staple food for >50% of the world’s population and plays an imperative part in the socio-economic development of many nations. Arsenic toxicity negatively affects rice growth and development, thereby causing significant yield losses [20]. In the literature, different studies have explored the role of BC or MT alone in mitigating the adverse effects of As. However, no information is available about the combined use of BC and MT in mitigating the adverse effects of As toxicity in rice. It was hypothesized that integrating BC and MT can more effectively decrease As toxicity. Therefore, this study was conducted with the following aims: (1) to determine the role of BC–MT combination on morpho-physiological and biochemical functioning and rice productivity. (2) To assess how BC and MT affect nutrient availability and As accumulation. (3) To determine how combined BC and MT affect endogenous MT synthesis, As speciation, and gene expression involved in As uptake, MT synthesis, and antioxidant activities.

## 2. Results

### 2.1. Biochar Characterization

**Figure 1:**
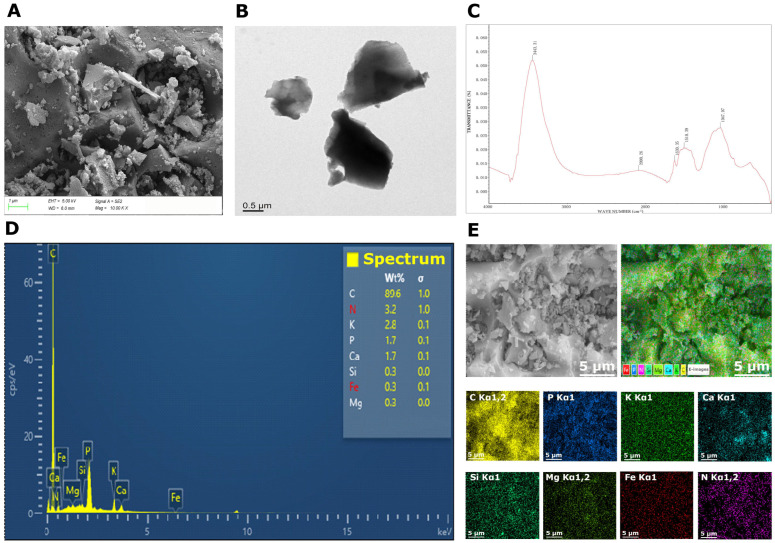
SEM (**A**), TEM (**B**), FTIR spectra of biochar (**C**), elements mapping (**D**) and EDS dispersion of biochar (**E**).

The properties of BC play a crucial role in remediating contaminated soil. Biochar made from coconut fiber showed a porous structure demonstrating its appreciable ability to absorb toxic metals (Figure 1). Biochar subjected to fourier transform infrared spectroscopy (FTIR) analysis showed peaks at 3443.31 (O-H), 2090.26 (C=O), 1630.35 (C=C), 1510.39 (C-O), and 1047.07 (C-H) cm^−1^ (Figure 1). The peak at 3443.31 cm^−1^ was expected from the O-H group, and the peak at 2090.26 was expected to be from the C=O group, while the peak recorded at 1630.35, 1510.39 and 1047.07 cm^−1^ corresponded to C=C, C-O and C-H groups (Figure 1) [21]. This indicates that BC contained significant amounts of carboxylic and carbonyl groups (Figure 1). The EDS analysis was also performed to confirm the presence of different elements in BC. Biochar contained a major portion of carbon and it also contained appreciable amounts of different nutrients, such as nitrogen, phosphorous, potassium, calcium, silicon, iron and magnesium, respectively (Figure 1).

### 2.2. Physiological Traits

**Figure 2:**
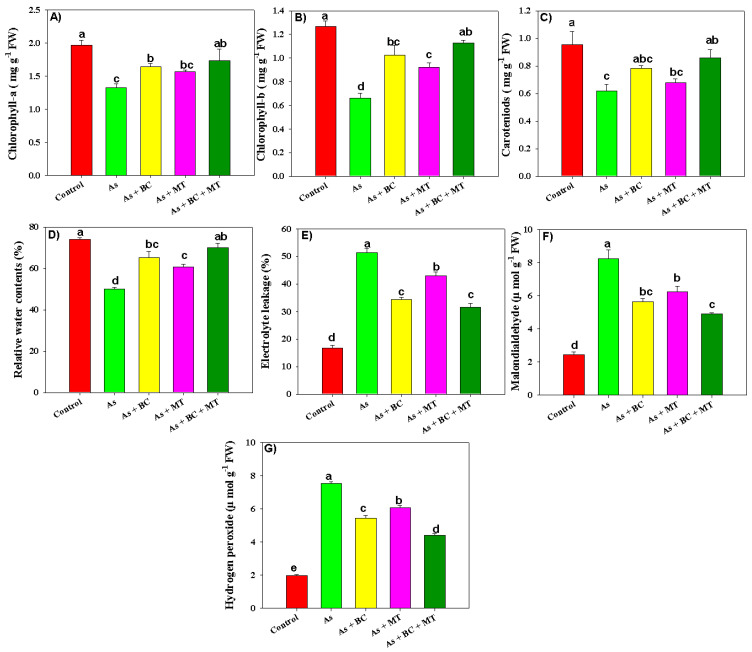
Effect of biochar and melatonin application on chlorophyll-a (**A**), chlorophyll-b (**B**), carotenoids (**C**), relative water contents (**D**), electrolyte leakage (**E**), malondialdehyde (**F**) and hydrogen peroxide (**G**) production in rice grown in As contaminated soil. The data is means (n = 3) and different letters with means show significance with ± SD at *p* ≤ 0.05.

Arsenic significantly decreased leaf chlorophyll and relative water contents (RWC) (Figure 2). Arsenic toxicity inhibited chl-a, chl-b, and carotenoid synthesis by 48.12%, 92.42% and 53.22%, respectively, compared to the control (Figure 2). Compared with the control, As decreased leaf RWC by 48% (Figure 2). In contrast, the BC and MT application caused a marked increase in both photosynthetic pigments and leaf RWC (Figure 2). Leaf chl-a, chl-b, carotenoid, and RWC contents were considerably increased by 30.07%, 71.21%, 38.70%, and 40%, respectively, under As stress with co-applied BC and MT (Figure 2). BC and MT alone also significantly increased the photosynthetic pigments and RWC; however, better results were seen with co-applied BC and MT (Figure 2).

### 2.3. Oxidative Markers and Antioxidant Activities

**Figure 3:**
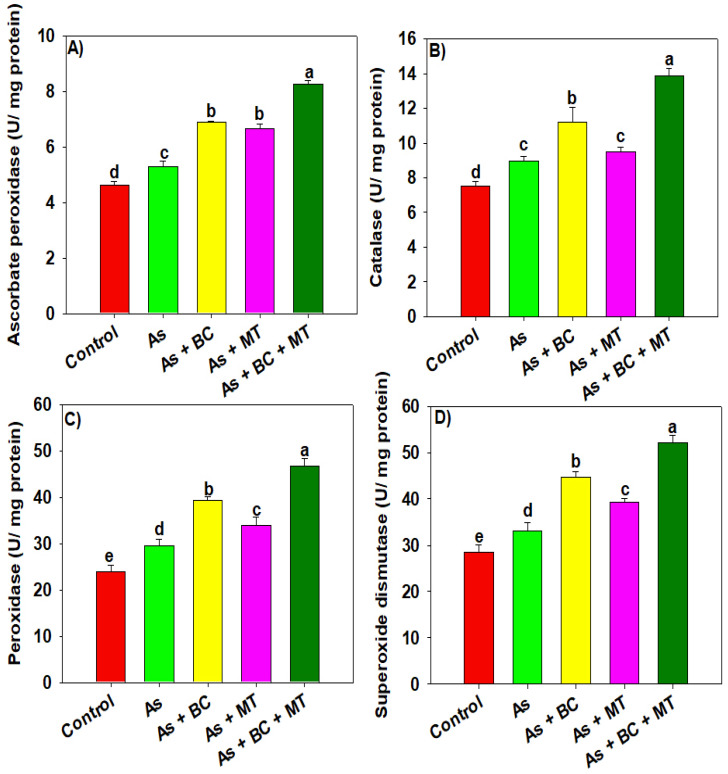
Effect biochar and melatonin on ascorbate peroxide (**A**), catalase (**B**), peroxidase (**C**) and super oxide dismutase (**D**) activities of rice plants grown in As contaminated soil. The data is means (n = 3) and different letters with means show significance with ± SD at *p* ≤ 0.05.

The results indicated that, compared with the control, As substantially increased oxidative markers (Figure 2). We found that As significantly increased electrolyte leakage (EL), malondialdehyde (MDA), and hydrogen peroxide (production by 206.81%, 238.25%, and 279.79%, respectively. The co-application of BC and MT, and their individual applications, significantly decreased oxidative markers; however, better results were observed with the integrative BC and MT application (Figure 1). Co-applying BC and MT decreased EL, MDA, and H_2_O_2_ production by 62.28%, 40.67%, and 75.66%, respectively, under As -polluted soil (Figure 2). The presented results depicted that antioxidant activity increased under As, indicating the plants activated their defense system to counter As toxicity. Nevertheless, combined BC and MT, as well as their individual application, also significantly increased all antioxidant activities, yet better results were observed with the combined BC and MT application (Figure 3). We witnessed an increase of 56.43%, 55.14%, 57.77%, and 57.52%, respectively, in ascorbate peroxidase (APX), catalase (CAT), peroxidase (POD), and superoxide dismutase (SOD) activities under As stress with co-applying BC and MT (Figure 3).

### 2.4. Osmolytes, Endogenous Melatonin and Phytochelatin Synthesis

**Figure 4:**
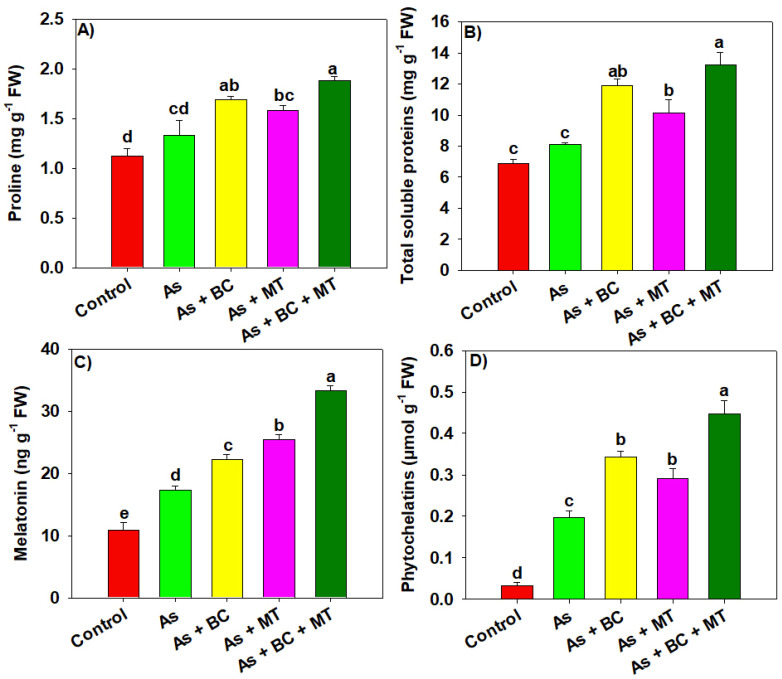
Effect of biochar and melatonin on proline (**A**), total soluble proteins (**B**), melatonin (**C**) and (**D**) phytochelatins synthesis of rice plants grown in As contaminated soil. The data is means (n = 3) and different letters with means show significance with ± SD at *p* ≤ 0.05.

The results showed that osmolytes and endogenous MT synthesis increased under As, compared to the control, indicating the potential of rice plants to increase osmolyte and MT synthesis to counter As toxicity (Figure 4). The synthesis of total soluble proteins (TSP), proline, and MT was increased by 17.27%, 18.75%, and 63.84%, respectively. All of the amendments also substantially enhanced the synthesis of osmolytes and endogenous MT; however, better results were seen with combined BC and MT (Figure 4). The results showed an increase of 63.86%, 41.35%, and 91.53% in TSP, proline, and endogenous MT synthesis with co-applied BC and MT (Figure 4). Different treatments significantly impacted the phytochelatins (PCs) synthesis. Co-applying BC + MT enhanced PCs synthesis by 125%, while BC and MT enhanced PCs synthesis by 70% and 45%, respectively, in As polluted soil (Figure 4).

### 2.5. Soil Properties and Arsenic Fractions

Biochar, MT, and their co-application caused a marked decrease in As availability (Table 1). Biochar combined with MT decreased soil As by 74.19%, whereas BC alone and MT alone decreased the soil As concentration by 46.26% and 21.45%, respectively (Table 1). All of the treatments also showed a significant impact on post-harvest soil properties (Table 1). The effect of MT was non-significant on soil pH; however, combined MT and BC showed a significant impact on soil pH. Arsenic toxicity also decreased the soil nitrogen (N), phosphorus (P), and potassium (K) availability by 53.16%, 51.56%, and 39.40%, respectively (Table 1). However, BC and MT significantly enhanced N, P, and K availability (Table 1). We found that co-applying BC and MT enhanced soil N, P, and K availability by 43.36%, 28.42%, and 29.26%, respectively, under As stress (Table 1).

**Figure 5:**
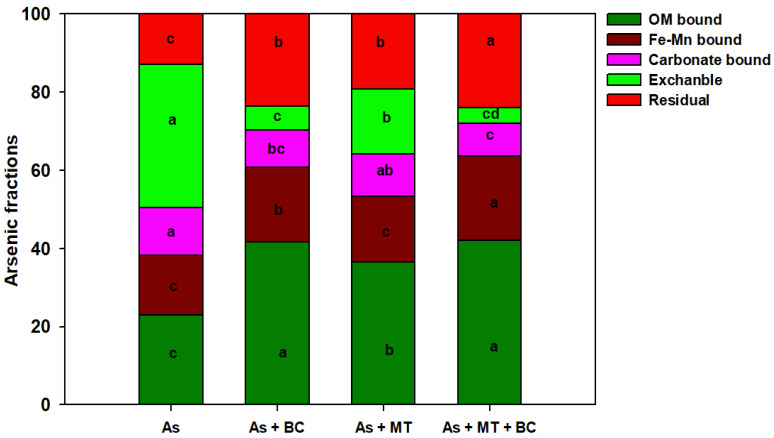
Effect of biochar and melatonin on different fractions of As in contaminated soil. The data is means (n = 3) of three replicates different letters with means show significance with ± SD at *p* ≤ 0.05.

Different treatments significantly impacted the soil As fractions (Figure 4). We found that maximum exchangeable As was found in As-contaminated soil, and all the treatments significantly decreased exchangeable As availability; however, better results were seen with co-applied BC and MT. Further, we also witnessed that As significantly increased carbonate-bound As, and BC and MT decreased its availability. The results also depicted that As decreased the availability of organic matter-bound, iron and manganese (Fe-Mn)-bound, and residual As. Biochar and MT, and their co-exposure, significantly enhanced these As fractions; nevertheless, more promising results were observed with combined BC and MT application (Figure 5).

### 2.6. Growth and Yield Traits and Element Accumulation in Rice Seedlings

Arsenic toxicity inhibited rice root length, and fresh and dry weights by 126.50%, 93.18%, and 124.16%, respectively, compared to the control (Table 2). All of the amendments significantly increased root growth, and taller roots with greater fresh and dry biomass were observed with co-applying BC and MT (Table 2). Arsenic toxicity also significantly decreased rice plant height, tillers, and grain weight. The combined use of BC and MT, and their individual use, significantly enhanced plant height, tiller production, and grain weight. Co-applied BC and MT enhanced plant height (PH), tillers, and thousand grain weight (TGW) by 22.69%, 22.22%, and 48.28%, respectively, compared to the control (Table 2). Arsenic toxicity also decreased biomass, grain yield, and harvest index (HI) by 33.28%, 35.17%, and 32.33%, respectively. The combined and individual applications of BC and MT significantly enhanced biomass, grain yield, and HI; however, better results were reported with co-applying BC and MT (Table 2). The results presented an increase of 26.06%, 31.43%, and 28.16%, respectively, in biomass, grain yield, and HI with co-applying BC and MT under As stress (Table 2).

**Figure 6:**
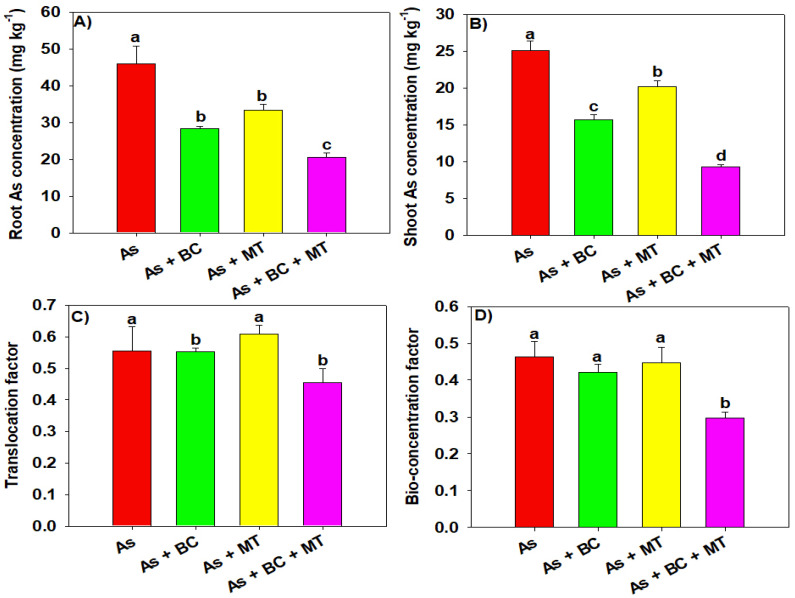
Effect of biochar and melatonin on root (**A**) and shoot (**B**) As concentrations, As translocation factor (**C**) and bio-concentration factor (**D**) of rice grown in As contaminated soil. The data is means (n = 3) and different letters with means show significance with ± SD at *p* ≤ 0.05.

The results indicated that maximum As accumulation in roots (46 mg kg^−1^) and shoots (25.15 mg kg^−1^) was observed under As. The application of BC and MT, and their combination, significantly decreased As in both roots and shoots; however, the lowest As concentrations in roots (20.53 mg kg^−1^) and shoots (9.26 mg kg^−1^) were observed with combined BC and MT application (Figure 6). The lowest TF and BCF of As were observed with co-applying BC and MT, compared to their individual applications (Figure 6). The results indicated that As significantly decreased N, P, K, calcium (Ca), and magnesium (Mg) accumulation in rice plants. In contrast, BC and MT enhanced nutrient accumulation, and maximum N, P, K, Ca, and Mg under As stress was seen with co-applied BC and MT, compared to their individual applications (Table 3).

### 2.7. Gene Expression

**Figure 7:**
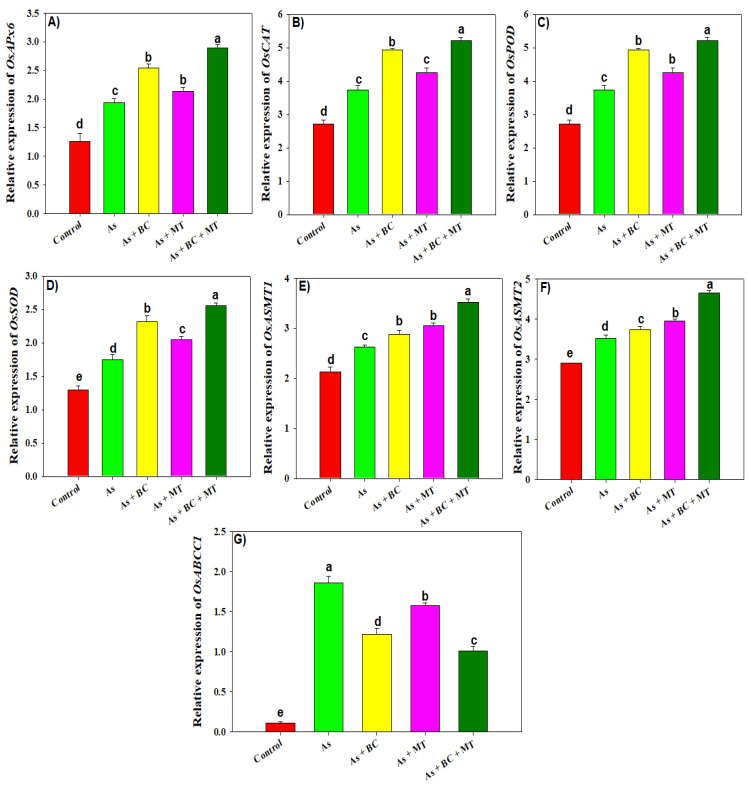
Effect of coconut shell biochar and melatonin on expression of *OsAPx6* (**A**), *OsCAT* (**B**), *OsPOD* (**C**), *OsSOD* (**D**), *OsASMT1* (**E**), *OsASMT2* (**F**) and *OsABCC1* (**G**) genes of rice plants grown in As contaminated soil. The data is means (n = 3) and different letters with means show significance with ± SD at *p* ≤ 0.05.

Different treatments showed a contrasting impact on the expression of antioxidant genes, MT synthesis genes, and As uptake gene expression (Figure 7). The lowest expression of all antioxidant genes was noted in the control, and under As stress, significantly enhanced expression of antioxidant genes was observed. The supplementation of BC, MT, and their co-exposure significantly enhanced gene expression (Figure 7). The co-application of BC and MT significantly enhanced *OsAPx6*, *OsCAT*, *OsPOD*, and *OsSOD* expressions by 38.46%, 48.96%, 39.30%, and 45.71%, respectively (Figure 7). We also observed that expression of *OsASMT1* and *OsASMT2* increased under As stress, and this was further increased with BC, MT, and their combined application. Biochar + MT increased *OsASMT1* and *OsASMT2* expression by 34.35% and 31.72%, respectively (Figure 7). The expression of the As uptake gene (*OsABCC1*) was significantly increased under As, which increased As uptake. However, BC, MT, and BC + MT significantly decreased the expression of *OsABCC1* by 52.45%, 29%, and 84.15%, respectively, under As stress (Figure 7).

### 2.8. Principal Component Analysis

**Figure 8:**
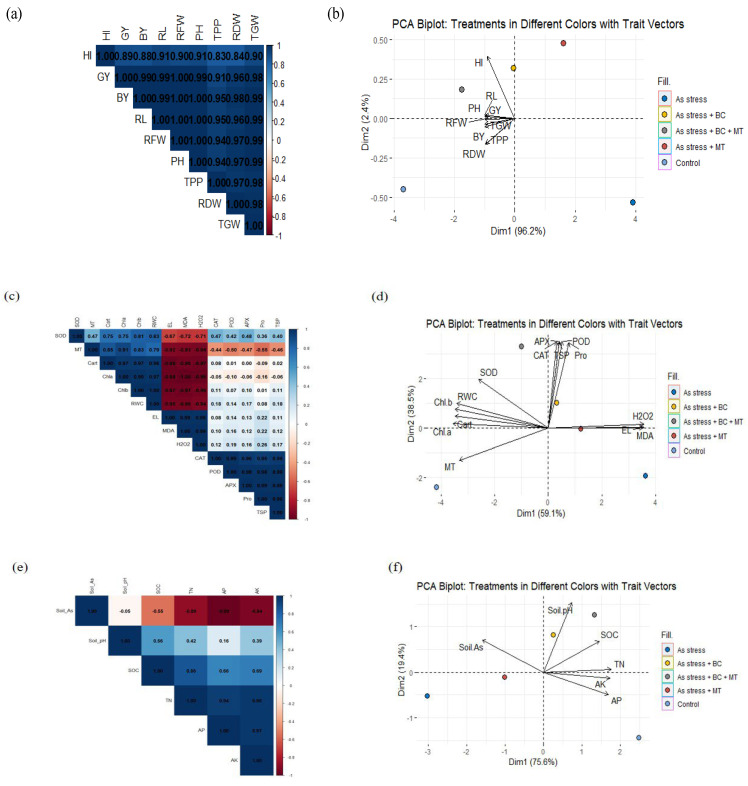

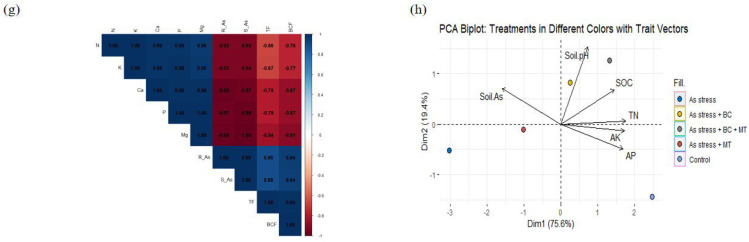
Principal component and correlation analysis for the impact of different treatments on growth (**a**,**b**), physio-biochemical traits (**c**,**d**), soil properties (**e**,**f**) and seedling nutrient concentration (**g**,**h**).

The first principal component (Dim1) accounted for 96.2% of the total variance, while the second component (Dim2) explained 2.4% (Figure 8). Arsenic stress alone clustered on the far right of the principal component analysis (PCA) space, indicating distinct trait expression patterns under stress conditions. In contrast, the control group appeared on the far left, indicating optimal trait expression in the absence of stress. Treatments involving BC and MT and their combinations (As + BC, As + MT, As +BC + MT) occupied intermediate positions, reflecting varying degrees of amelioration of stress effects (Figure 8). Trait vectors revealed that GY, BY, TPP, and TGW were the most influential contributors to Dim1, with their orientation indicating a positive association with the control and ameliorated treatments. Conversely, the As stress treatment showed a negative association with most of the measured traits, highlighting the detrimental impact of arsenic exposure on growth and yield-related parameters (Figure 8).

In case of physiological and biochemical traits the first two principal components (Dim1 and Dim2) accounted for 59.1% and 38.5% of the total variance, respectively. There was a strong correlation with oxidative stress markers like MDA, H_2_O_2_, and EL, suggesting an intensified stress response. In contrast, the control treatment (purple point) clustered on the far left and showed positive associations with traits, such as chlorophylls, carotenoids, RWC, and SOD, indicating an optimal physiological status. BC + MT (grey point), were grouped closer to the control, suggesting successful mitigation of arsenic toxicity. This exhibited positive associations with antioxidant enzymes and osmolyte accumulation, indicating enhanced stress tolerance (Figure 8).

PCA analysis of soil parameters revealed that the first two principal components (Dim1 and Dim2) explained 75.6% and 19.4% of the variance, respectively. As stress treatment (red point) and arsenic-only condition (blue point) were located on the negative side of Dim1, indicating high soil arsenic levels and a stressed, nutrient-deficient soil profile. In contrast, treatments with biochar clustered on the positive side of Dim1, aligned closely with available phosphorous (AP), available potassium (AK), total nitrogen (TN), soil organic carbon (SOC), and soil pH. This pattern illustrates that the combined treatment significantly enhanced soil fertility while reducing arsenic bioavailability.

### 2.9. Correlation Between Different Traits

The control treatment (purple point) also exhibited a strong association with nutrient-rich vectors, confirming optimal soil conditions in the absence of arsenic stress. The PCA biplot (Figure 4B) explained 75.6% of the total variation on Dim1 and 19.4% on Dim2 for seedling nutrients accumulation. As stress + BC + MT treatment appeared on the positive side of Dim1 and aligned with nutrient vectors (TN, AK, AP, SOC), suggesting that biochar and microbial amendments not only reduced arsenic uptake but also enhanced nutrient assimilation. The control treatment also clustered favorably, reinforcing the negative impact of arsenic stress and the restorative potential of the combined treatment (Figure 8).

The Pearson correlation matrix revealed strong and significant positive correlations among all of the traits studied under different treatments. Grain yield showed a strong positive correlation with BY (BY) (r = 0.99), RL (RL) (r = 0.99), and TPP (r = 0.91), indicating that these traits contribute substantially to grain yield under the tested conditions. Notably, shoot traits, such as PH, RFW, and RDW, also exhibited high positive correlations (r > 0.94) with one another, suggesting coordinated growth responses across the shoot–root continuum (Figure 8). The oxidative stress indicators, such as MDA and H_2_O_2_, were negatively correlated with all other traits. MDA showed very strong negative correlations with Cart (r = −0.98), chl-a (r = −0.99), and RWC (r = −0.96), implying increased membrane damage in conditions where pigment concentration and water status were compromised. Electrolyte leakage, an indicator of cell membrane injury, also showed strong negative correlations with photosynthetic and water-related traits (Figure 8).

Soil arsenic content was negatively correlated with key soil nutrients, particularly AP (r = −0.99), AK (r = −0.94), TN (r = −0.89), and SOC (r = −0.55), indicating that As depletes essential soil nutrients. Conversely, SOC showed strong positive correlations with TN (r = 0.86), AP (r = 0.66), and AK (r = 0.69), underscoring the importance of organic matter in sustaining soil fertility. Soil pH exhibited weak correlations with most variables, with slight positive associations observed with SOC (r = 0.56) and TN (r = 0.42), suggesting that pH-increasing amendments may marginally enhance soil fertility. Root shoot arsenic are strongly negatively correlated with seedling P (r = −0.97 and −0.98, respectively), Mg (r = −0.99 and −1.00), and K (r = −0.93 and −0.94). These correlations indicate that arsenic accumulation hinders nutrient uptake and assimilation in rice plants. Additionally, the translocation factor (TF) and bioconcentration factor (BCF) show negative associations with nutrient elements (especially Mg, Ca, and K) but positive correlations with R_As and S_As (r ≥ 0.88). This suggests that higher arsenic uptake and translocation lead to reduced nutrient availability in plant tissues (Figure 8).

## 3. Discussion

Rice is a major cereal crop cultivated globally, and As accumulation in rice is a serious challenge [22]. Thus, it is crucial to develop effective remediation strategies to present the As accumulation in rice and its entry into human food chain [23]. In this study, As toxicity significantly decreased rice growth and productivity by inhibiting chlorophyll synthesis (Figure 2), root growth, and nutrient uptake [24]. Plants grown under As also faced morphological and physiological impairment due to excessive MDA and H_2_O_2_ production, inhibited chlorophyll synthesis [25], and poor nutrient uptake [26]. All of these changes significantly reduced plant growth and yield [27,28]. Biochar and MT synergistically enhanced rice productivity by increasing soil fertility, and decreasing As availability [29,30]. Combining BC and MT increases chelation, immobilization, and complexation of As, which reduces As availability, leading to better growth and yield [13,31]. Biochar and melatonin also improves root growth, which facilitates better water (Figure 1) and nutrient uptake (Table 3), resulting in significant increase in growth [32]. Biochar and MT considerably enhances endogenous MT synthesis, and antioxidant defense, which protects rice plants from the damaging impacts of As, thereby enhancing rice productivity [31]. Additionally, BC + MT enhances the phytochelatin synthesis, which might promote the As accumulation vacuole and reduces its availability. This in turn enhances rice growth and yield.

Photosynthesis is an important process for carbon fixation and biomass production, and chlorophyll plays a crucial role in this process [33]. Arsenic significantly decreases chlorophyll synthesis by increasing ROS production, which disrupts chloroplast membranes, degrades the photosynthetic apparatus and ribulose-1,5-bisphosphate carboxylase/oxygenase (RuBisCO) activity, leading to reduction in photosynthetic efficiency [34,35]. These results are consistent with earlier findings indicating that As degrades chlorophyll synthesis and plant photosynthetic efficiency [36]. Biochar and MT application significantly enhanced chlorophyll synthesis, by increasing endogenous MT synthesis antioxidant activities, and decreasing As availability. This protected the photosynthetic apparatus from the damaging impacts of As, thus ensuring better chlorophyll synthesis. Both BC and MT also increased Mg uptake, and its accumulation, which plays a key role in chlorophyll synthesis [33,37]. The improved root growth after BC and MT application enhanced nutrient and water uptake resulting in better chlorophyll synthesis and plant growth [38]. Arsenic toxicity significantly decreased leaf RWC by decreasing root growth, root hydraulic conductivity, and cell turgor pressure. Both BC and MT significantly increased leaf RWC by increasing root growth, which enhanced the water uptake, leading to better RWC.

Arsenic increased ROS production, which damaged membrane integrity, as indicated by an increase in MDA production (Figure 2) [39,40]. Biochar and MT supplementation significantly enhanced all antioxidant activities (APX, CAT, POD, and SOD), which countered As-induced oxidative stress and resulted in less MDA and H_2_O_2_ production [40]. CAT and POD convert H_2_O_2_ into non-toxic compounds, while SOD converts superoxide into H_2_O_2_ [23]. Interestingly, BC and MT significantly enhanced genes expression (*OsAPx6*, *OsCAT*, *OsPOD*, and *OsSOD*) involved in antioxidants. This increased the antioxidant activities, which react with ROS and convert these oxidative molecules into neutral and non-toxic compounds [41,42]. Osmolyte accumulation plays a crucial role in maintaining plant water status under stress conditions [43]. We noted that TSP, proline, and MT synthesis increased under As stress, which was further increased with BC and MT supplementation. This increased proline and MT biosynthesis maintained osmotic and protected rice plants from the damaging impacts of As [44]. Biochar and MT increased the endogenous MT by increasing the expression of *OsASMT1* and *OsASMT2* involved in MT biosynthesis. The increase in MT synthesis enhanced antioxidant activities and reduced As uptake and accumulation, leading to better growth and yield. Phytochelatins (PCs) play a crucial role in mitigating the heavy metal toxicity owing to their ability to chelate metal ions with -SH groups [45]. We observed that PCs synthesis increases under As toxicity, which was further increased with BC and MT. This aligns with earlier studies reporting that MT application enhanced PCs synthesis under HM [46]. Phytochelatins bind the As to -SH grounds and form the stable PCs-As complexes leading to reduction in As availability. Furthermore, PCs also indirectly mitigate the oxidative damages by decreasing HM availability and their interference with essential nutrients leading to better growth under HM toxicity [47].

Nutrient uptake plays a crucial role in plant growth under stress conditions. Arsenic stress decreased N, P, K, Ca, and Mg accumulation in rice compared to the control. Arsenic damages plant roots and inhibits the functioning of nutrient channels present in roots, which diminish nutrient uptake and their accumulation in plant tissues [48]. Nevertheless, BC and MT increased N, P, K, Ca, and Mg uptake and accumulation. Biochar increases nutrient availability and their uptake, thus increasing nutrient accumulation in plant organs [48]. Biochar and MT significantly enhanced root growth, and subsequently, root hydraulic conductivity and water retention, which helped in nutrient absorption from the soil, leading to increased nutrients accumulation [49]. Potassium plays a vital role in gas exchange, enzyme activities, and RuBisCO activity, while calcium regulates stomatal movements and electron transport. Therefore, BC and MT mediated an increase in uptake of these nutrients caused a marked increase in plant growth [50,51,52]. The expression of *OsABCC1* was increased under As stress, which enhanced As uptake and accumulation in rice plants. Conversely, BC, MT, and their combination, decreased the expression of *OsABCC1*, which decreased As uptake and its accumulation in plant tissues. This aligns with earlier results indicating that MT mitigates As toxicity by decreasing As uptake and accumulation through suppression of gene expression involved in As uptake [53]. The expression of antioxidant genes increased with BC and MT, which increased the antioxidant activities and protected the rice plants from toxic impacts of As. These findings are the same as earlier studies reporting that BC and MT increased antioxidant gene expression to counteract heavy metal toxicity [54,55].

We observed that BC increased soil pH, which was linked with the alkaline nature of BC and the presence of hydroxides and carbonates in BC, which had a liming effect [56]. This might also be linked with an increase in the secretion of alkaline compounds from plants after MT application, and an increase in soil microbial activities, which release alkaline substances, thus raising soil pH [57,58]. Arsenic toxicity significantly decreased soil N, P, and K availability. This decrease in nutrient availability with arsenic (As) can be attributed to competition between As and other nutrients, which reduces nutrient absorption and leads to a decrease in nutrient availability [59]. Furthermore, As negatively impacts soil microbial communities involved in nutrient cycling, and this disruption reduces the release of nutrients, leading to a reduction in nutrient availability [19]. Additionally, it imposes negative impacts on plant roots (Table 1), which damage the cellular membranes and impair the ability of roots to absorb nutrients [60], resulting in a reduction in soil nutrient availability. We also observed that BC with MT decreased soil exchangeable As availability. This was linked with the fact that BC + MT increased soil pH and decreased the solubility of exchangeable As, causing its immobilization and complexation, thus reducing its availability. It is well reported that BC fixes the As and transfers As into forms with better migration ability, which in turn reduces the As availability [61,62]. The increase in soil pH enhances the complexation and immobilization of As, thereby decreasing its availability. Generally, As occurs in weakly charged species in acidic soils; an increase in soil pH enhances its deprotonation into negatively charged oxyanions, which increases its interaction with soil minerals. Therefore, an increase in soil pH causes As complexation, resulting in a substantial reduction in As availability [61,62]. Biochar has higher surface area, porosity, and functional groups (Figure 1), which effectively immobilize As and reduce its availability [63]. The higher surface area of BC also adsorbs the As through electrostatic interactions and sorption, leading to a reduction in As availability [64]. Furthermore, functional groups present on the BC surface also cause complexation of As, which reduces its mobility and bioavailability in soil solution [65,66]. We also observed that foliar-applied MT significantly reduced As uptake and accumulation in plant tissues, suggesting MT might work as a barrier that inhibits As uptake and its translocation from roots to shoots [19,54]. Vacuoles are the main sites for detoxification of heavy metals [67]. Different studies witnessed that As is sequestered into vacuoles in the form of As-phytochelatin conjugate [68]. We observed that MT enhanced the phytochelatin synthesis, which promoted the As accumulation in the vacuole, thus reducing As transport from roots to shoots [46,54]. Melatonin chelates As, and forms stable complexes with As, thus reducing its availability [46,54]. This process increases the As adsorption to organic matter and manganese oxides (Figure 5), limiting its uptake plants. Therefore, it is suggested that MT represses the As absorption and its movement in rice plants. We found that BC and MT increased the availability of OM-bound, Fe (Fe_2_As) and Mn ((Mn_3_(AsO_4_)_2_)-bound and residual As forms. Biochar increases Fe and Mn availability, which bind As, thus decreasing its availability [69]. A biochar-mediated increase in Fe availability increases the fixation of As, thus decreasing its availability in soil [70]. It has been documented that higher Fe content in soil increases As fixation and decreases its utilization rate [71]. Biochar + MT enhances residual and OM-bound fractions of As by increasing soil OM availability.

## 4. Materials and Methods

### 4.1. Experiment Site and Biochar Preparation

This study was performed at Jiangxi Agricultural University, China, from May 2024 to July 2024. The soil was taken from a 0–30 cm soil layer of the experimental site. The soil was sieved, debris was removed, and pots were filled with 10 kg of dry soil. The soil was identified as acidic, with a pH of 5.5, along with available phosphorus and potassium contents of 33.43 and 114.33 mg kg^−1^, respectively, and a total nitrogen content of 1.62 g kg^−1^. The coconut fiber was purchased from a local market and its pyrolysis was performed at 600 °C for 4 h; thereafter, BC was sieved (2 mm) to estimate different properties. The biochar had a pH of 9.98, with a cation exchange capacity (CEC) of 74.3 cmol kg^−1^ and a carbon content of 588 g kg^−1^. The concentration of carbon in biochar was determined by a potassium dichromate external heating technique with concentrated H_2_SO_4_. Furthermore, CEC of biochar was measured by modified ammonium acetate compulsory displacement method [72,73]. 

### 4.2. Experimental Details

The experiment contained the following treatments: control, As stress (100 mg kg^−1^), As stress + BC (2%), As stress + MT (100 µM), and As stress + BC + MT. Sodium arsenate (NaAsO_2_) was used to achieve the desired As concentration. Biochar was applied at the rate of 20 g per kg dry soil to achieve a rate of 2%. Arsenic salt was mixed with soil and stabilized for two months under 70% of field capacity. Thereafter, the soil from the pots was taken out, BC was mixed with the soil, and the pots were filled again. Five seedlings (25 days old) were transplanted into each experimental pot, and a 2–3 cm water level was maintained in each pot during the growing period. Melatonin was applied as a foliar spray after 20 days of transplanting until the plants became fully wet. The foliar spray was performed with a hand sprayer until the plants became fully wet. The study was conducted using a completely randomized design with three replications.

### 4.3. Determination of Chlorophyll Synthesis, Leaf Water Contents and Oxidative Markers

For this, 0.5 g of freshly collected leaves were extracted with 80% acetone at 0–4 °C. The samples were centrifuged (10,000 rpm) for five minutes, and absorbance was noted at 663, 645, and 470 nm to determine chl a, b, and carotenoids [74]. The rice leaf samples were collected and weighed (FW). These samples were then soaked in water for 24 h and weighed again (TW). The samples were dried (65 °C) and weighed again (DW), and RWC was measured as: (FW − DW)/(TW − DW) × 100. In the case of electrolyte leakage (EL), freshly collected leaf samples were heated in water (40 °C) for half an hour, and electrical conductivity (EC: EC1) was recorded. Then, samples were again soaked in water at 40 °C for 10 min, and a second reading (EC2) was noted, and EL was estimated as: EL% = (EC1/EC2) × 100. The standard procedures of [75] were utilized to determine leaf MDA by using thiobarbituric acid (TBA). Briefly, 0.5 g of fresh leaves was homogenized in 2 mL of 50 mM cooled phosphate-buffered saline (PBS: pH 7.8). Then, the extract was centrifuged (6000× *g*) for 15 min. After centrifugation, 0.5 mL of the supernatant was combined with 1 mL of 20% (*w*/*v*) trichloroacetic acid and 0.5% TBA. The mixture was incubated at 95 °C for 15 min, and then cooled down. It was centrifuged again (8000× *g*) for 10 min, and absorbance was noted at 450, 532, and 600 nm to estimate MDA concentration. In the case of H_2_O_2_, 0.5 g of freshly collected leaves were extracted with 0.1% tri-chloro acetic acid (TCA: 1 mL) solution and centrifuged (8000 rpm). Thereafter, the supernatant was carefully collected and mixed with 10 mL of potassium phosphate buffer (PPB) and 1 mL of 1 M potassium iodide, and the absorbance of the resulting solution was measured at 600 nm [76].

### 4.4. Determination of Leaf Osmolytes, Endogenous Melatonin and Antioxidants

For measuring leaf proline, fresh samples were taken and homogenized in 3% sulfosalicylic acid solution and centrifuged (10,000 rpm) for 10 min. The mixture was heated for 1 h at 100 °C after mixing with glacial acetic acid (2 mL) and acid ninhydrin (2 mL). The mixture was then placed at room temperature, and after cooling, toluene (2 mL) was added, and absorbance was taken at 520 nm [77]. To estimate TSP, 0.5 g of freshly collected leaves were ground in PPB (5 mL) to obtain an extract. The centrifugation of the extract was performed for 15 min at 14,000 rpm. Thereafter, 2 mL of Bradford reagent was added to the extract, and absorbance was measured at 595 nm [78]. To measure endogenous MT, rice leaf samples were collected and ground, then mixed with chloroform (5 mL). This mixture was centrifuged (10,000 × *g*) for 10 min. The chloroform was then evaporated at room temperature, and the concentration of MT was measured with HPLC [79]. For measuring APX activity, we prepared a 3 mL reaction mixture containing 100 mM PPB, ethylenediaminetetraacetic acid (EDTA) (0.1 mM), ascorbic acid (0.3 mM), H_2_O_2_ (0.06 mM), and enzyme extract (100 μL). Absorbance was recorded at 290 nm for measuring APX activity. For measuring catalase activity, the reaction mixture containing PPB (50 mM), H_2_O_2_ (10 mM), and enzyme extract (2 mM) was prepared. CAT activity was determined by measuring H_2_O_2_ degradation at 240 nm [80]. For estimating peroxidase (POD) activity, we prepared a mixture containing 0.75% H_2_O_2_ solution (0.05 mL), 0.25% guaiacol solution (0.25 mL), 0.995 mL of PBS (100 mM), and 0.05 mL enzyme extract, and absorbance was noted at 460 nm to estimate POD [81]. Superoxide dismutase (SOD) was assessed by measuring absorbance at 560 nm following the protocols of [82] and expressed in U mg^−1^ protein.

### 4.5. Tissue Nutrient and Arsenic Concentration

Plant samples were milled to powder and then subjected to digestion on a hot plate using hydrochloric acid (HCl) and nitric acid (HNO_3_) (1:2). After digestion, the samples were filtered and diluted with distilled water to a final volume [83]. Later, the concentration of N and P in plant samples was measured using the Kjeldahl and spectrophotometer procedures. Further, the concentration of calcium and magnesium was measured using an Atomic Absorption Spectrophotometer, and K was estimated using a flame photometer [84]. For measuring As concentration, powdered rice samples (0.5 g) were digested using nitric and hydrochloric acids (3:1). The samples were carefully digested until acid fumes turned white. Then, deionized water was added to dilute them, and they were filtered. Arsenic concentration was determined by atomic absorption spectrometry. Further, the translocation factor (TF) and bio-concentration factor (BCF) were measured using the formula suggested by [85].

TF = As in shoots/As concentration in roots

BCF = As content in roots/As concentration in soil

### 4.6. Determination of Soil Properties

The samples were collected after harvesting, and all plant material and debris were removed. The pH of the soil was measured by pH meter in a soil-to-water ratio of 1:5 [57], whereas soil N was measured with the Kjeldahl procedure as suggested by [86]. Furthermore, soil AP was determined with spectrophotometer and flame photometer methods as suggested by [87] and concentration of AK was measured by ammonium acetate extraction technique, respectively, as suggested by [88]. For measuring soil As, samples were digested with nitric and hydrochloric acids (3:1). The samples were carefully digested until the acid fumes turned white. Then, samples were diluted by adding deionized water and filtered. Later, As content was estimated by atomic absorption spectrometry. The different fractions of As in soil samples were measured using the procedures of [89]. Different forms of As, including residual, OM-bound, Fe-Mn bound, carbonate-bound, and exchangeable, were determined.

### 4.7. Agronomic Parameters

After 90 days of transplanting, the plants were harvested to measure root and shoot length and their biomass. The tillers were manually counted, and spike lengths were measured and averaged. The harvested plants from each pot were weighed for measuring biomass yield, and later, grains were threshed and weighed to measure grain yield. Further, the harvest index was calculated as the ratio of grain to biomass yields.

### 4.8. RNA Preparation and Gene Expression Analysis

The rice leaf samples were collected, and total RNA was extracted using an RNA extraction kit from Takara, China. For synthesis of cDNA, 2.5 µL of RNA was reverse-transcribed using the HiScript^®^ III RT SuperMix kit. For performing real-time PCR, 1 µg RNA was reverse-transcribed into cDNA using the FastKing gDNA Dispelling RT SuperMix Kit. Further, qRT-PCR was performed using the SuperReal PreMix Plus (SYBR Green) Kit (Tiangen; FP205–2). The expression of genes was calculated by the method of [90]. Additionally, details about the primers used in gene expression analysis are given in Supplementary Table S1.

### 4.9. Determination of Phytochelatin Synthesis

The freshly collected leaves (1 g) were ground with liquid nitrogen. Thereafter, the leaves were mixed with 1 mL ice-cool extraction buffer solution containing 6.3 mM diethylene triamine pentaacetic acid (DTPA) and 0.1% trifluoroacetic acid solutions. Then, the supernatant was collected and centrifuged (12,000× *g*) for 10 min and mixed with monobromobimane. Later, samples were filtered using a 45 µm nylon syringe filters and subjected to high-performance liquid chromatography HPLC to determine the concentration of phytochelatin [91,92]. The authentic standards (e.g., PC2 and PC3) provided by Sigma-Aldrich were used to determine the phytochelatin. The calibration curves for the standards were prepared, and the peaks in the samples were compared with the curves for quantification.

### 4.10. Statistical Analysis

All of the collected data were analyzed using analysis of variance (ANOVA) with Statistix 8.1^®^ software. Before subjecting to ANOVA, the data were subjected to Normal distribution and homogeneity of variances. Furthermore, significant differences between means were compared by the Honestly Significant Difference (HSD) test at *p* < 0.05. The figures used in this study were generated using SigmaPlot-10 and R-studio v.1.4.1564.

## 5. Conclusions

Arsenic toxicity led to a considerable decrease in rice growth and productivity, due to impaired plant functioning, reduced nutrient availability, and increased arsenic availability. Integrated biochar and melatonin synergistically mitigated arsenic toxicity via increasing osmolyte synthesis, antioxidant defense, and melatonin synthesis, and reducing oxidative stress markers. This was also associated with increased soil nutrient availability, antioxidant and melatonin synthesis gene expression, reduced expression of the arsenic uptake gene, and arsenic accumulation in plant tissues. Biochar and melatonin also converted arsenic into more stable forms, hence reducing its uptake and accumulation, leading to better growth. Thus, using biochar and melatonin together could be a beneficial strategy to enhance rice productivity in arsenic-polluted soils. The combination of biochar and melatonin significantly enhanced most of the observed traits as compared to their single application. In most cases, the observed improvement exceeded the sum of their individual impacts, indicating the synergistic effect of combined biochar and melatonin application. However, long-term investigations are needed to validate the effects of this practice in different soil and climatic conditions. Furthermore, molecular investigations are required to elucidate the synergistic mechanisms mediated by biochar and melatonin to counter arsenic toxicity. The combination of melatonin + biochar costs around 8–10 USD for this experiment. The cost of this combination could vary in different areas; therefore, it is suggested that the author should include the cost of this combination in future studies.

## Figures and Tables

**Table 1:** Effect of coconut shell biochar and melatonin on soil properties of rice grown in As contaminated soil.

Treatments	Soil As (mg kg^−1^)	Soil pH	TN (g kg^−1^)	AP (mg kg^−1^)	AK (mg kg^−1^)
Control	-	5.62 ± 0.021 c	1.72 ± 0.07 a	53.70 ± 1.81 a	128.63 ± 2.36 a
As	54.46 ± 2.21 a	5.58 ± 0.026 c	1.12 ± 0.03 d	35.43 ± 0.86 d	92.27 ± 1.72 d
As + BC	37.23 ± 1.78 c	5.94 ± 0.028 b	1.49 ± 0.04 bc	42.73 ± 1.25 bc	109.54 ± 1.24 c
As + MT	45.43 ± 3.27 b	5.60 ± 0.041 c	1.38 ± 0.02 c	40.30 ± 0.74 c	98.37 ± 2.05 d
As+ BC + MT	31.27 ± 0.78 c	6.12 ± 0.021 a	1.61 ± 0.05 ab	45.50 ± 0.90 b	119.26 ± 2.80 b

The data is means (n = 3) and different letters with means show significance with ± SD at *p* ≤ 0.05. As: arsenic, TN: total nitrogen, AP: available phosphorous, AK: available potassium. BC: biochar, MT: melatonin.

**Table 2:** Effect of coconut shell biochar and melatonin on growth and yield traits of rice grown in As contaminated soil.

Treatments	RL (cm)	RFW (g)	RDW (g)	PH (cm)	TPP	TGW (g)	BYP (g)	GYP (g)	HI (%)
Control	35.04 ± 2.55 a	14.74 ± 0.47 a	5.38 ± 0.09 a	101.67 ± 2.87 a	11 ± 1.25 a	28.32 ± 0.85 a	156.81 ± 2.58 a	18.06 ± 0.81 a	21.61 ± 1.94 a
As	15.47 ± 0.86 d	7.63 ± 0.43 c	2.40 ± 0.13 d	79.33 ± 2.49 d	9 ± 0.94 b	16.63 ± 1.23 d	117.65 ± 2.62 c	13.36 ± 0.79 d	16.33 ± 0.35 b
As + BC	26.40 ± 0.70 b	11.20 ± 0.82 b	3.45 ± 0.19 c	91.00 ± 2.45 bc	10 ± 0.47 ab	22.69 ± 0.47 bc	135.67 ± 2.49 b	15.67 ± 0.86 bc	20.57 ± 0.90 a
As + MT	21.10 ± 0.95 c	9.67 ± 0.41 bc	3.10 ± 0.09 c	86.00 ± 1.41 cd	10 ± 0.49 ab	19.88 ± 1.26 c	127.33 ± 4.11 bc	14.71 ± 0.38 cd	19.83 ± 0.45 ab
As+ BC + MT	31.31 ± 0.82 a	13.33 ± 0.90 a	4.19 ± 0.16 b	97.33 ± 1.70 ab	11 ± 1.25 ab	24.66 ± 0.63 b	148.31 ± 3.23 a	17.56 ± 0.30 ab	20.93 ± 1.27 a

The data is means (n = 3) and different letters with means show significance with ± SD at *p* ≤ 0.05. RL: root length, RFW: root fresh weight, RDW: root dry weight, PH: plant height, TPP: tillers/plant: TGW: 1000 grain weight, BYP: biomass yield/pot, GYP: grain yield/pot, HI: harvest index, BC: biochar, MT: melatonin, As: arsenic.

**Table 3:** Effect of coconut shell biochar and melatonin on concentration of essential nutrients in rice seedlings.

Treatments	Nitrogen (mg kg^−1^)	Phosphorous (mg kg^−1^)	Potassium (mg kg^−1^)	Calcium (mg kg^−1^)	Magnesium (mg kg^−1^)
Control	42.00 ± 1.73 a	25.50 ± 0.78 a	61.70 ± 1.24 a	66.90 ± 1.28 a	62.07 ± 1.67 a
As	26.30 ± 0.80 d	14.33 ± 0.17 e	33.70 ± 1.36 e	37.10 ± 1.61 d	36.58 ± 0.85 e
As + BC	36.20 ± 0.50 bc	20.20 ± 0.82 c	51.97 ± 0.56 c	51.50 ± 0.82 c	47.81 ± 1.16 c
As + MT	34.00 ± 0.72 c	17.67 ± 0.41 d	46.83 ± 0.43 d	49.80 ± 4.80 c	43.03 ± 1.24 d
As+ BC + MT	39.70 ± 1.28 ab	22.63 ± 0.48 b	56.83 ± 1.25 b	57.50 ± 0.82 b	54.07 ± 1.33 b

The data is means (n = 3) and different letters with means show significance with ± SD at *p* ≤ 0.05. BC: biochar, MT: melatonin, As: arsenic.

## Data Availability

Data will be made available on request.

## Supplementary Materials

The following supporting information can be downloaded at: https://www.mdpi.com/article/10.3390/plants14152453/s1, Table S1: List of primers used for gene expression analysis.
