# Supplementary material for: Biochar and Melatonin Partnership Mitigates Arsenic Toxicity in Rice by Modulating Antioxidant Defense, Phytochelatin Synthesis, and Down-Regulating the Transporters Involved in Arsenic Uptake
Source: Plants (Basel). 2025 Aug 7;14(15):2453. doi: 10.3390/plants14152453 (PMC12349230; doi:10.3390/plants14152453)
Supplement: Supplementary file 1 [file plants-14-02453-s001.zip › plants-3764003-supplementary.pdf]

Table S1: List of primers used for gene expression analysis

Gene	Details of primers
OsAPx6-F	CCTTCACCTGCGGAACATCT
OsAPx6-R	AGCACAGCATCAGTAGGCAG
OsCAT-F	GCCACGAAGGACTTGACTGA
OsCAT-R	GAGATCCAGATGCCACGGAG
OsPOX-F	GAAGGGTTGATGTTGCTGCC
OsPOX-R	TCGGCGTTCTTTGATGTCCT
OsSOD-F	TACGGGTAGGGCACTGAACA
OsSOD-R	CTCCTTTCCGGCAGGATTGT
OsASMT1-F	ATATTCCATGACGCGGGCTT
OsASMT1-R	TGGGTAAACCTCGATGACGG
OsASMT2-F	CGCATCCTCGTCGGTGATAA
OsASMT2-R	ATGAGCCATCTCGAAGAGCG
OsABCC1-F	AACAGTGGCTTATGTTCCTCAAG
OsABCC1-R	AACTCCTCTTTCTCCAATCTCTG
Actin-F	CATTGGTGCTGAGCGTTTCC
Actin-R	CCCGCAGCTTCCATTCCTAT
